# Endoscopic Management of a Right Maxillary Sinus Mucocele With Palatal and Medial Wall Erosion

**DOI:** 10.7759/cureus.102657

**Published:** 2026-01-30

**Authors:** Retaj Alawadhi, Sood Alsairefi, Jassem Bastaki, Marwan Al-Qunaee

**Affiliations:** 1 Department of Otolaryngology, Head and Neck Surgery, Kuwait Institute for Medical Specialization (KIMS), Kuwait City, KWT; 2 Plastic and Reconstructive Surgery Unit, General Surgery Deparment, Jaber Al-Ahmad Hopspital, Kuwait City, KWT; 3 Department of Histopathology, Al-Sabah Hospital, Kuwait City, KWT; 4 Department of Otolaryngology, Zain Hospital, Kuwait City, KWT

**Keywords:** endoscopic marsupialization, endoscopic sinus surgery, maxillary sinus mucocele, medial wall erosion, odontogenic cyst, odontogenic epithelium, palatal erosion, paranasal sinus lesion

## Abstract

A mucocele is a benign, mucus-filled cystic lesion resulting from obstruction of the sinus ostium, leading to gradual mucus accumulation and remodeling of the surrounding bony walls. A mucocele affecting the maxillary sinus is known as a maxillary sinus mucocele. Although less common than frontal or ethmoidal mucoceles, large maxillary mucoceles may extend to adjacent structures, producing ophthalmic or dental manifestations. In severe cases, they can cause facial swelling, bony erosion, and midfacial disfigurement, sometimes mimicking aggressive or neoplastic pathology.

Here, we present a case of a 27-year-old male with a history of multiple sclerosis who presented with right facial swelling, facial pain, nasal obstruction, and nasal discharge. Clinical examination showed a tender, fluctuant swelling over the right maxilla, and computed tomography demonstrated a large expansile cystic lesion occupying the right maxillary sinus. The patient underwent endoscopic marsupialization and resection under general anesthesia, with excellent postoperative recovery. Histopathology demonstrated a cystic cavity lined by attenuated simple squamous epithelium merging with native respiratory mucosa, indicating a maxillary sinus mucocele with an associated odontogenic epithelial component.

This case highlights the importance of considering a maxillary sinus mucocele in the differential diagnosis of unilateral facial swelling and nasal obstruction. It underscores the diagnostic challenge it poses when mimicking chronic rhinosinusitis or odontogenic cysts, and emphasizes the crucial role of early radiologic evaluation and multidisciplinary collaboration in achieving accurate diagnosis and preventing complications.

## Introduction

Paranasal mucoceles develop as an obstructive complication of chronic sinusitis, tumors, or trauma of the paranasal sinuses. The maxillary sinus, the largest of the paranasal sinuses, is pyramid-shaped and located within the maxillary bone, with its ostium (drainage opening) located in the middle meatus of the nasal cavity; it is in close proximity to the orbit superiorly, the palate and dentition inferiorly, and the nasal cavity medially [1]. This anatomical positioning explains why expansile lesions like mucoceles can lead to pressure-induced bone resorption (remodeling due to chronic internal pressure from mucus accumulation) or, if infected, transform into a pyocele (containing pus) via bacterial superinfection. Fronto-ethmoidal mucoceles are the most common, whereas maxillary and sphenoid sinus mucoceles are rare. When a mucocele becomes infected, it may transform into a pyocele containing pus cells.

Accumulation of mucus within these cystic lesions leads to progressive expansion and potential complications depending on their origin and direction of growth [1]. If a maxillary sinus mucocele enlarges sufficiently to cause bone resorption, it may produce ophthalmic or dental symptoms, and in rare cases, midfacial swelling or deformity [2]. Due to the close anatomical relationship between the sinuses and the orbit, ocular manifestations, such as proptosis, diplopia, or visual disturbance, are common, especially in ethmoid and sphenoid mucoceles [3].

The most frequent predisposing factors include chronic infection, allergic rhinitis, trauma, and previous surgery, although many cases are idiopathic [4]. Ostial obstruction refers to the blockage of the sinus drainage pathway (ostium), often leading to mucus retention, while Schneiderian mucosa is the pseudostratified ciliated columnar epithelium lining the paranasal sinuses. Diagnosis is primarily radiologic: computed tomography (CT) delineates bony expansion and thinning, while magnetic resonance imaging (MRI) better characterizes soft-tissue signal and protein content, differentiating mucoceles from neoplasms or inflammatory masses [5,6]. Cone-beam CT (CBCT) can further quantify maxillary sinus volume and anatomic relationships, aiding preoperative planning for sinus lesions.

Endoscopic sinus surgery (ESS) has become the gold standard for treatment, offering a minimally invasive, well-tolerated approach with excellent outcomes [7]. This case demonstrates a maxillary sinus mucocele with palatal and medial wall erosion managed endoscopically, illustrating the value of prompt recognition and multidisciplinary care.

This case contributes to the literature by highlighting a rare presentation of a maxillary mucocele with combined palatal and medial wall erosion and an odontogenic component in a young patient with multiple sclerosis. It aligns with case series emphasizing the efficacy of endoscopic management in preventing complications and underscores the clinical importance of early intervention to avoid misdiagnosis as neoplasms or chronic rhinosinusitis [2,3,5].

## Case presentation

A 27-year-old Kuwaiti male with a history of multiple sclerosis (MS), obsessive-compulsive disorder, and Tourette syndrome was referred to the Otorhinolaryngology Department at Al-Sabah Hospital for evaluation of a right maxillary lesion incidentally detected on MRI during hospitalization for urinary symptoms related to MS.

He reported progressive right facial fullness, intermittent facial pain, nasal obstruction, and occasional nasal discharge over 6 months. He denied diplopia, epistaxis, or visual changes. There was no history of sinonasal surgery, trauma, or chronic sinusitis.

On examination, there was a mild right-sided facial asymmetry with fullness over the maxilla, characterized by a tender, fluctuant swelling measuring approximately 3 cm in diameter, without lymphadenopathy or skin changes. Endoscopy revealed bulging of the right lateral nasal wall and narrowing of the nasal cavity. Intraoral examination showed a firm, non-ulcerated swelling of the right vestibule. Cranial nerves were intact, and no ophthalmologic or dental abnormalities were noted.

**Figure 1:**
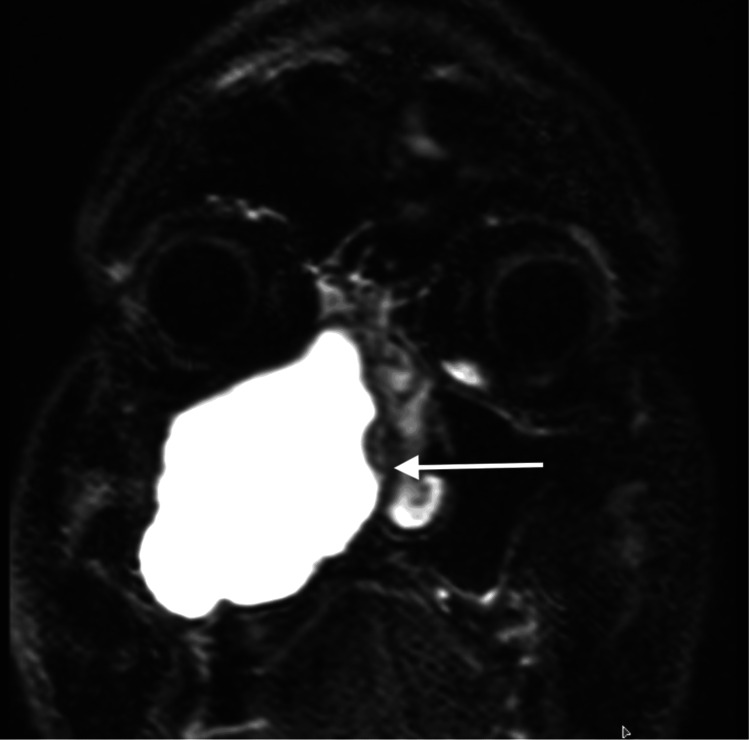
Coronal MRI of the paranasal sinuses Coronal T1-weighted MRI showing a well-defined expansile hyperintense lesion occupying the right maxillary sinus, with medial wall erosion and upward displacement of the orbital floor. MRI: magnetic resonance imaging

MRI of the paranasal sinuses showed a well-defined expansile hyperintense lesion filling the right maxillary sinus, with medial wall erosion and upward displacement of the orbital floor (Figure 1). 

**Figure 2:**
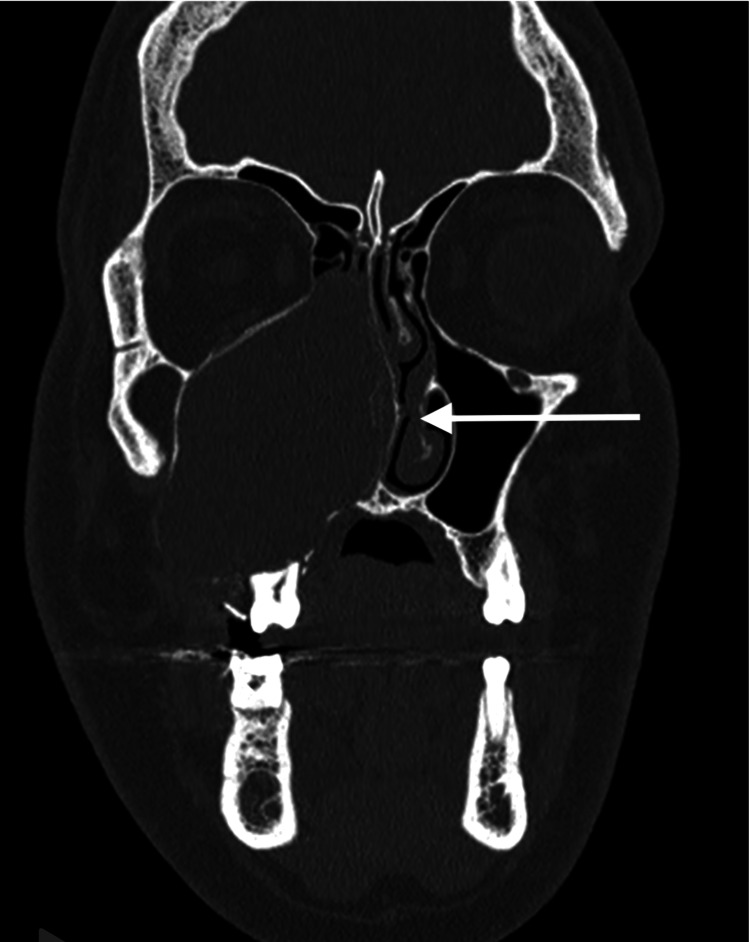
Coronal CT of the paranasal sinuses (bone window) Coronal CT scan demonstrating complete opacification and expansion of the right maxillary sinus with thinning and remodeling of the medial and palatal bony walls, extending toward the nasal cavity. CT: computed tomography

CT demonstrated a cystic expansile lesion with thinning and outward bowing of the sinus walls, extending toward the nasal cavity without orbital or intracranial invasion (Figure 2).

**Figure 3:**
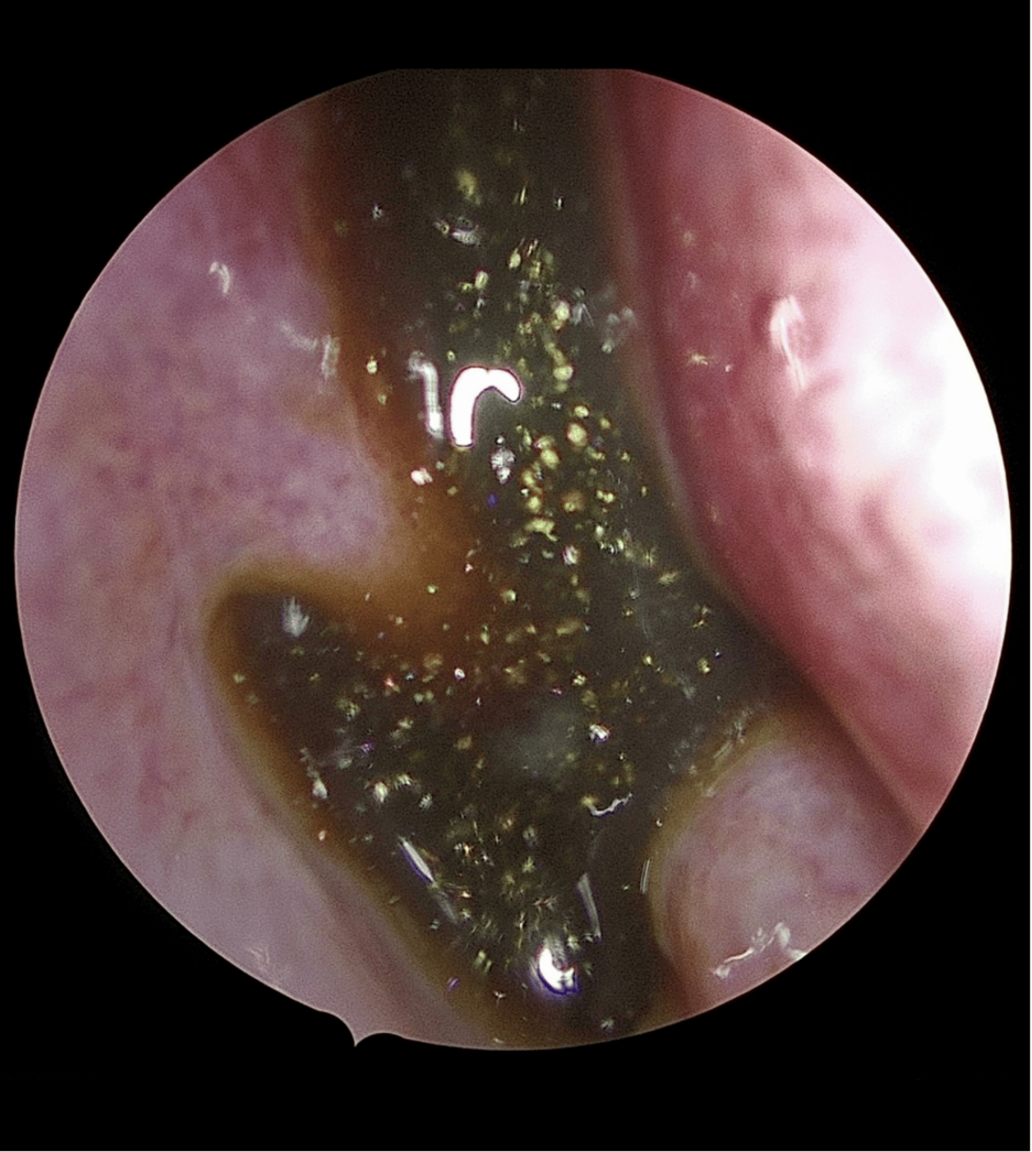
Intraoperative endoscopic view Endoscopic intraoperative image showing drainage of thick mucoid material from the right maxillary sinus during marsupialization.

The patient underwent endoscopic marsupialization and resection under general anesthesia. Intraoperatively, an expansile cyst was seen eroding the right medial maxillary sinus wall and extending inferiorly toward the palatal bone. A retained/impacted tooth was visualized at the floor of the right maxillary sinus adjacent to the lesion. A large amount of thick brown mucoid fluid was drained and sent for culture and cytology, and the cyst was widely opened into the nasal cavity. Hemostasis was achieved, and a right Merocel pack was placed (Figure 3). Culture and cytology results were negative for infection or malignancy.

**Figure 4:**
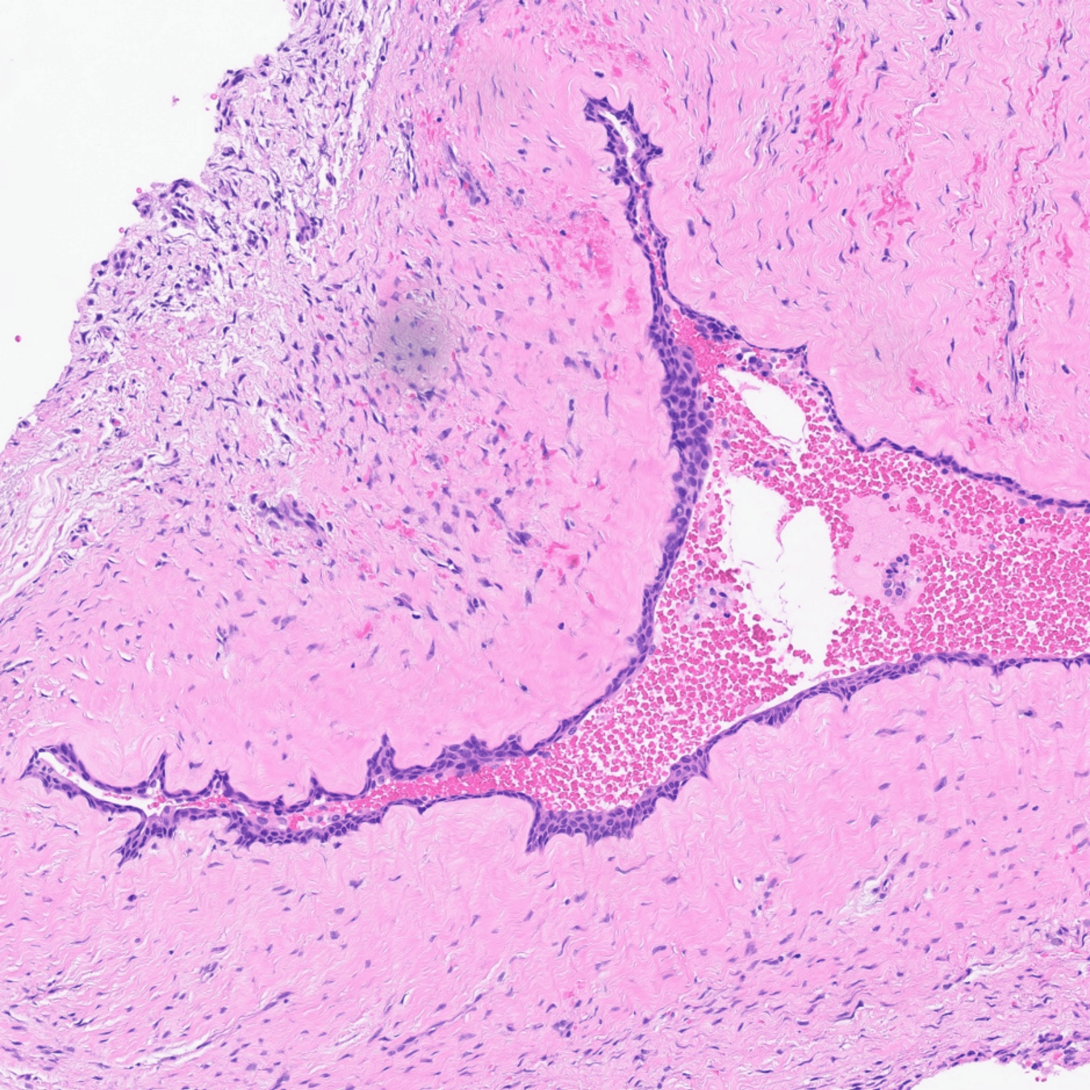
Histopathology (odontogenic component) High-magnification photomicrograph showing H&E stained attenuated simple squamous epithelial lining, likely representing an odontogenic cyst component secondarily involving the maxillary sinus. H&E: Hematoxylin & Eosin

**Figure 5:**
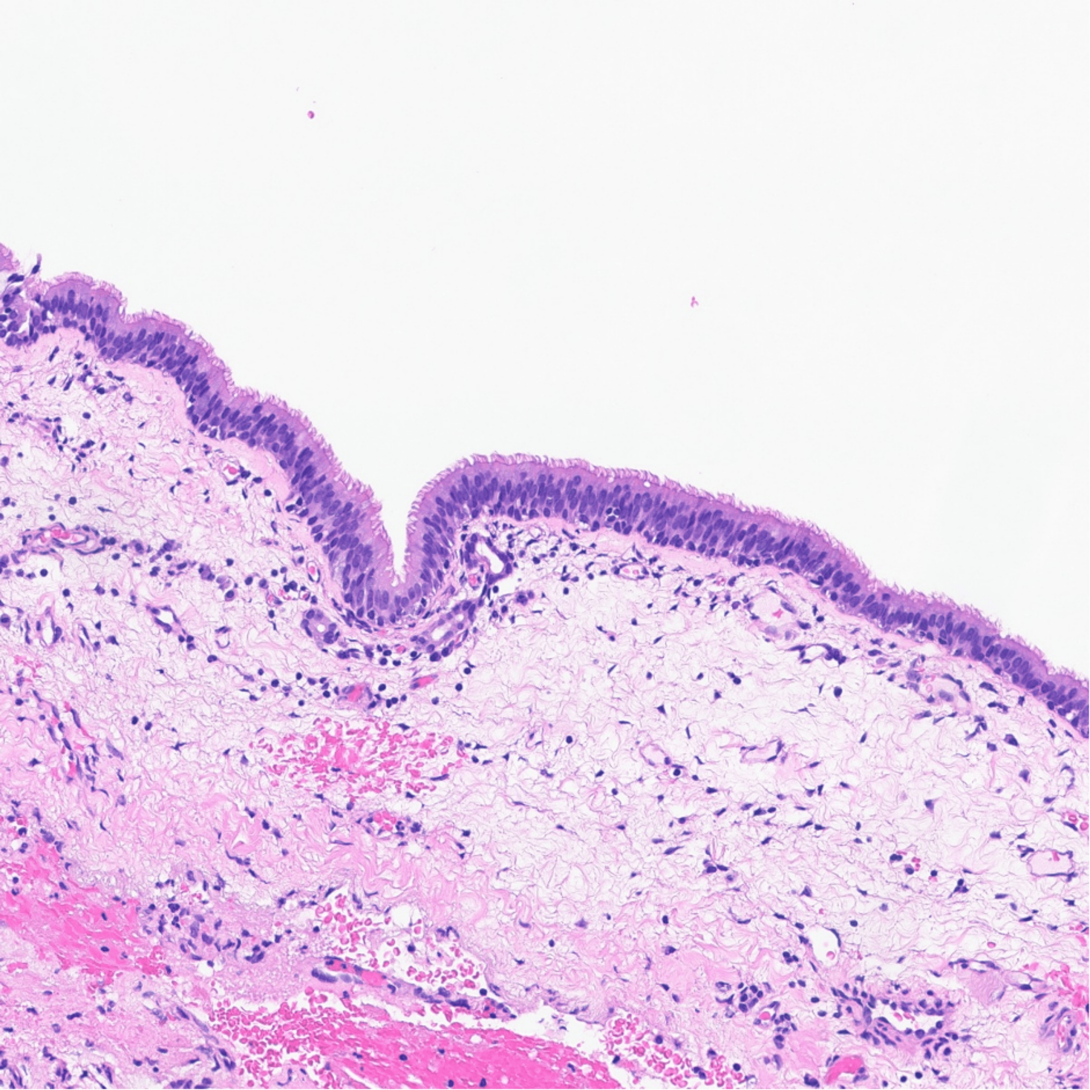
Histopathology (respiratory epithelium) High-magnification photomicrograph showing H&E stained pseudostratified ciliated columnar respiratory epithelium (Schneiderian mucosa) lining the cystic lesion, consistent with a maxillary sinus mucocele. H&E: Hematoxylin & Eosin

Histopathology revealed a large cystic lesion lined by attenuated simple squamous epithelium in continuity with pseudostratified ciliated columnar (respiratory) mucosa. The squamous component likely represents odontogenic cyst lining that has secondarily merged with the native Schneiderian mucosa (Figures 4, 5). 

These findings confirmed the diagnosis of a maxillary sinus mucocele with an associated odontogenic epithelial element. The postoperative course was uneventful, and the patient remained asymptomatic at follow-up with a patent sinus ostium. Follow-up at 3 months showed a patent ostium on endoscopy, with no symptoms or recurrence.

## Discussion

Maxillary sinus mucoceles are benign expansile cysts resulting from ostial obstruction and mucus retention [1]. They constitute less than 10% of all paranasal sinus mucoceles [2,3]. Clinical manifestations are variable, ranging from nasal obstruction and facial swelling to dental pain or orbital displacement, depending on the lesion’s size and direction of expansion [4].

In this patient, imaging revealed both medial and palatal wall erosion, consistent with chronic pressure remodeling. The presence of an unerupted or impacted tooth at the sinus floor, documented intraoperatively, likely contributed to ostial blockage and chronic mucus retention. Odontogenic elements have been reported in mucoceles that arise adjacent to unerupted or ectopic teeth, with epithelial transition occurring as odontogenic rests merge with native Schneiderian mucosa. This mechanism explains the attenuated simple squamous epithelium noted on histopathology in continuity with the respiratory mucosa in the present case [5].

Imaging is critical for diagnosis, with CT scanning being the preferred method to evaluate bone remodeling, while MRI offers better soft-tissue detail, typically showing high T2 signal intensity and variable T1 signals depending on protein concentration [6]. CT showed Hounsfield units of 10-20 HU (consistent with mucus), while MRI demonstrated hyperintensity on T1/T2 without contrast enhancement. This imaging combination allows differentiation from cystic neoplasms, odontogenic cysts, or chronic fungal sinusitis [7]. Differential diagnoses included primary odontogenic cysts, chronic rhinosinusitis, and neoplasm, which were excluded based on hybrid epithelium, lack of inflammatory signs, and absence of histopathological atypia, respectively. Clinical reasoning combined progressive, expansile, and mucus-producing features to confirm a benign diagnosis, ruling out malignancy.

Histopathologic examination demonstrated a cystic lesion lined by attenuated simple squamous epithelium merging with pseudostratified ciliated columnar respiratory mucosa. This dual epithelial pattern has been described in mucoceles associated with odontogenic cysts, where odontogenic epithelium becomes incorporated into the expanding sinus lesion [3,5]. Importantly, no atypia or dysplasia was identified, confirming the lesion’s benign, non-neoplastic nature.

Endoscopic marsupialization has replaced the Caldwell-Luc procedure as the preferred treatment, providing adequate drainage and ventilation while minimizing morbidity [8-10]. Recurrence rates following ESS are exceptionally low (<5%) when wide marsupialization is achieved [9]. Our patient’s postoperative course was favorable, consistent with findings from large clinical series reporting rapid symptom resolution and minimal recurrence [10-13].

Although mucoceles are classically lined by respiratory epithelium, the presence of attenuated simple squamous epithelium in this case suggests secondary involvement by odontogenic cystic epithelium. Similar hybrid epithelial patterns have been described in mucoceles that expand into regions containing odontogenic rests. Identifying this subtle microscopic variation is crucial to prevent misclassification as a primary odontogenic cyst, highlighting the necessity of thorough histological examination for sinonasal cystic lesions.

**Table 1:** Summary of key findings This table summarizes preoperative imaging measurements, intraoperative findings, pathology, and follow-up status.

Aspect	Details
Preoperative Imaging	Lesion size: 4.5 × 3.2 × 3.8 cm; Medial wall thinning: ~2 mm; Palatal erosion: 5 mm; HU: 10-20
Intraoperative	~50 mL thick mucoid fluid drained; Retained or impacted tooth at sinus floor
Pathology	Hybrid epithelium (squamous merging with respiratory); No atypia
Follow-up (3 months)	Patent ostium; Asymptomatic; No recurrence

Key learning points

In the evaluation and management of conditions presenting with unilateral facial swelling or nasal obstruction, several critical principles emerge from clinical practice and evidence-based guidelines. Patients exhibiting these symptoms, particularly when unilateral, should undergo prompt cross-sectional imaging, such as CT or MRI, regardless of the absence of severe accompanying symptoms. This approach is essential to identify underlying structural abnormalities, inflammatory processes, or potential malignancies that may not be evident on clinical examination alone, thereby facilitating early detection and appropriate intervention.

Furthermore, achieving diagnostic accuracy is significantly improved through collaborative interdisciplinary efforts. Close coordination among ear, nose, and throat (ENT) surgeons, radiologists, and pathologists allows for integrated interpretation of clinical findings, imaging results, and histopathological data, leading to more precise diagnoses and tailored treatment plans.

Finally, timely endoscopic intervention plays a pivotal role in restoring physiological function. By establishing effective drainage pathways early in the disease course, this minimally invasive technique helps maintain normal sinonasal anatomy, reduces the risk of secondary infections, and minimizes complications such as bone erosion or destruction.

## Conclusions

This case highlights the diagnostic challenge of differentiating a maxillary sinus mucocele from odontogenic or neoplastic lesions. Timely CT and MRI imaging, supported by histopathologic confirmation, is essential for accurate diagnosis. Endoscopic marsupialization remains the treatment of choice, offering excellent outcomes, low morbidity, and low recurrence when complete drainage is achieved. Multidisciplinary collaboration is vital to ensure optimal management and long-term, disease-free recovery.
